# Managing Pneumonia Due to Rare Non-Fermenting Gram-Negative Bacteria: Epidemiology, Risk Factors and Therapeutic Strategies

**DOI:** 10.3390/antibiotics15050465

**Published:** 2026-05-04

**Authors:** Alessandro Capone, Francesca Gavaruzzi, Valentina Antonelli, Claudia Rotondo, Samir Al Moghazi, Emanuela Caraffa, Pierangelo Chinello, Carla Fontana, Stefania Cicalini

**Affiliations:** 1 Systemic and Immune Depression-Associated Infections Unit, National Institute for Infectious Diseases “Lazzaro Spallanzani”, IRCCS, 00149 Rome, Italy; alessandro.capone@inmi.it (A.C.); francesca.gavaruzzi@inmi.it (F.G.); samir.almoghazi@inmi.it (S.A.M.); emanuela.caraffa@inmi.it (E.C.); pierangelo.chinello@inmi.it (P.C.); 2 Microbiology and Biobank Unit, National Institute for Infectious Diseases “Lazzaro Spallanzani”, IRCCS, 00149 Rome, Italy; valentina.antonelli@inmi.it (V.A.); claudia.rotondo@inmi.it (C.R.); carla.fontana@inmi.it (C.F.)

**Keywords:** pneumonia, non-fermenting Gram-negative bacteria, *Sphingomonas* spp., *Aeromonas* spp., *Roseomonas* spp., *Achromobacter* spp., *Burkholderia* spp., *Pandoraea* spp., *Elizabethkingia* spp., *Kerstersia gyiorum*

## Abstract

Pneumonia remains a leading cause of morbidity and mortality worldwide, with bacterial pathogens contributing significantly to its burden. While *Pseudomonas aeruginosa* and *Acinetobacter baumannii* complex are well-recognized non-fermenting Gram-negative bacteria (NFGNB) causing severe pneumonia, particularly in healthcare settings, an expanding array of other, rarer NFGNB species is increasingly implicated. These species include, but are not limited to, *Achromobacter* spp., *Ochrobactrum* spp., *Burkholderia* spp., *Aeromonas* spp., *Roseomonas* spp., *Elizabethkingia* spp., *Chryseobacterium* spp. *Alcaligenes* spp., *Ralstonia* spp., *Cupriavidus* spp., *Sphingomonas* spp., *Rhizobium* spp., *Empedobacter* spp., and *Brevundimonas* spp. In this article we aim to provide a focused review of the contemporary epidemiology and specific risk factors for pneumonia caused by this diverse group of rare NFGNB, explicitly excluding *P. aeruginosa*, *Stenotrophomonas maltophilia*, and *A. baumannii*. We seek to delineate the emerging patterns of pneumonia associated with *Achromobacter* spp., *Burkholderia* spp., *Aeromonas* spp., *Roseomonas* spp., *Elizabethkingia* spp., *Pandoraea* spp., *Sphingomonas* spp., and *K. gyiorum.* Moreover, we discuss antimicrobial treatment strategies for pneumonia caused by rarer NFGNB including *Ochrobactrum* spp., *Chryseobacterium* spp., *Alcaligenes* spp., *Ralstonia* spp., *Cupriavidus* spp., *Rhizobium* spp., *Empedobacter* spp., and *Brevundimonas* spp. A deeper understanding of these specific epidemiological trends and risk factors is important for guiding precise diagnostic approaches, informing antimicrobial stewardship programs, and developing targeted infection prevention and control strategies with the aim of mitigating the impact of these challenging pathogens in the clinical setting.

## 1. Introduction

Pneumonia remains a leading cause of morbidity and mortality worldwide, with bacterial pathogens contributing significantly to its burden. While *Pseudomonas aeruginosa* and *Acinetobacter baumannii* complex are well-recognized non-fermenting Gram-negative bacteria (NFGNB) causing severe pneumonia, particularly in healthcare settings, an expanding array of other rarer NFGNB species is increasingly implicated [1,2]. These species include, but are not limited to, *Achromobacter* spp., *Ochrobactrum* spp., *Burkholderia* spp., *Aeromonas* spp., *Roseomonas* spp., *Elizabethkingia* spp., *Chryseobacterium* spp., *Alcaligenes* spp., *Ralstonia* spp., *Cupriavidus* spp., *Sphingomonas* spp., *Rhizobium* spp., *Empedobacter* spp., and *Brevundimonas* spp. [3,4].

Although *P. aeruginosa* multi-drug resistant (MDR) and carbapenem-resistant *A. baumannii* complex remain major drivers of hospital pneumonia, rare NFGNB represent an additional challenge because their resistance is often intrinsic, heterogeneous, and poorly predictable at the genus level. Many of these organisms exhibit reduced outer-membrane permeability, active efflux, and/or chromosomal β-lactamases, resulting in frequent mismatch between empiric regimens and true susceptibility. Consequently, early species-level identification (e.g., matrix-assisted laser desorption/ionization time-of-flight mass spectrometry (MALDI-TOF MS), with molecular confirmation when needed) can support earlier therapy refinement while minimun inhibition concentration (MIC)-based AST results are pending [5].

Because many rare NFGNB exhibit inherent and unpredictable resistance profiles, early identification at the species level is essential for timely optimization of antimicrobial therapy. In routine practice, MALDI-TOF MS has significantly reduced identification times and could provide reliable results within minutes of bacterial growth, compared to the several hours or days required by conventional biochemical methods or molecular approaches such as 16S rRNA sequencing. While highly accurate, especially for rare or difficult-to-identify organisms such as NFGNB, antimicrobial susceptibility testing (AST) is slower and more resource-intensive than MALDI-TOF MS. However, AST still requires additional incubation time, typically 16–24 h for standard phenotypic methods. This creates a clinically relevant time lag between species identification and the availability of susceptibility results, during which therapeutic decisions must be based on known resistance profiles. Therefore, early identification may allow for rapid restriction or intensification of therapy while awaiting MIC-based confirmation.

Despite the growing clinical recognition of these diverse rare NFGNB in pneumonia, comprehensive epidemiological data and understanding of their specific risk factors remain largely fragmented. Much of the existing literature either focuses on the more common NFGNB or broadly categorizes these less frequently encountered pathogens, obscuring their unique epidemiological niches and predisposing factors [6]. However, recent research has begun to shed more light on the individual characteristics of pneumonia caused by these organisms. For instance, *Achromobacter* spp. are increasingly identified in patients with underlying lung diseases, including non- cystic fibrosis (CF) bronchiectasis, and are often associated with prior antibiotic exposure and device-related infections [7,8]. Similarly, *Elizabethkingia* spp., though rare, are linked to severe infections, particularly in neonates and immunocompromised adults, characterized by intrinsic resistance to many common antimicrobials [9]. Other NFGNB, such as *Chryseobacterium indologenes* and *Aeromonas* species, typically found in environmental sources, are being reported in hospital-acquired pneumonia, especially in patients with comorbidities and indwelling medical devices [10,11]. Recent literature has identified *Kerstersia gyiorum* as a clinically relevant pathogen in chronic infections, particularly in patients with underlying neurodegenerative or vascular diseases [12,13]. Identification is challenging due to phenotypic similarities with other non-fermenters, necessitating MALDI-TOF MS and 16S rRNA gene sequencing.

In many laboratories, MALDI-TOF MS is widely available and can often provide organism identification within the same working day, soon after growth. In contrast, 16S rRNA gene sequencing is usually carried out in specialist or reference laboratories and can take longer due to batching and workflow constraints.

Notably, *K. gyiorum* exhibits variable antimicrobial susceptibility; resistance to fluoroquinolones and colistin has been reported, while susceptibility to β-lactams and aminoglycosides remains. These findings highlight the importance of comprehensive microbiological investigations and personalised treatment strategies for managing infections caused by rare NFGNB.

This review provides a focused synthesis of the contemporary epidemiology and specific risk factors for pneumonia caused by rare NFGNB. It explicitly excludes *P. aeruginosa*, *S. maltophilia* and the *A. baumannii* complex. These organisms are referenced only for brief contextual comparison and are not reviewed in detail. We seek to delineate the emerging patterns of pneumonia associated with *Achromobacter* spp., *Burkholderia* spp., *Aeromonas* spp., *Roseomonas* spp., *Elizabethkingia* spp., *Pandoraea* spp., *Sphingomonas* spp., and *K. gyiorum.* Moreover, we discuss antimicrobial treatments for pneumonia caused by rarer NFGNB, including *Ochrobactrum* spp., *Chryseobacterium* spp., *Alcaligenes* spp., *Ralstonia* spp., *Cupriavidus* spp., *Rhizobium* spp., *Empedobacter* spp., and *Brevundimonas* spp.

To maintain coherence, we prioritise information that directly informs clinical decision-making, presenting pathogens using a harmonised, clinically oriented structure. Topics that are not directly related to NFGNB pneumonia or that do not affect diagnostic or therapeutic choices are summarised briefly or omitted. A deeper understanding of these specific epidemiological trends and risk factors is vital for guiding precise diagnostic approaches, informing antimicrobial stewardship programs, and developing targeted infection prevention and control strategies to mitigate the impact of these challenging pathogens in the clinical setting [14,15,16,17].

## 2. Microbiology

NFGNB comprise a diverse group of aerobic, non-spore-forming bacilli that cannot ferment carbohydrates. Instead, they derive energy by oxidizing simple carbohydrates. These organisms are widely distributed in the environment as saprophytes, typically inhabiting moist ecosystems such as water, soil, and plants and can replicate under adverse environmental conditions. Additionally, some NFGNB are recognized as members of the healthy human gut microbiota [18]. While their environmental presence is well documented, their increasing role in clinical settings has drawn significant attention in recent years. Over the past few decades, NFGNB have increasingly emerged as significant nosocomial pathogens. This trend can be attributed to their remarkable ability to persist in hospital environments, such as sinks, respirators, nebulizers, dialysate, saline, catheters, and the surfaces of various medical devices. These observations support the need for robust infection-prevention measures, local surveillance, and antimicrobial stewardship to limit device- and environment-related transmission of rare NFGNB in hospital settings [19]. Recent surveillance data from the European Centre for Disease Prevention and Control indicate a steady rise in infections caused by NFGNB, particularly in intensive care units (ICUs) and among immunocompromised patients. These organisms are now recognized as key contributors to healthcare-associated infections, often linked to multi-drug resistance and environmental persistence [20]. Furthermore, their intrinsic AMR phenotypes pose additional challenges in treatment. As a result, this group of microorganisms is increasingly acknowledged as a leading cause of difficult-to-treat infections, particularly in vulnerable patient populations. These high-risk groups include neutropenic individuals, patients in ICUs, and those with CF, where NFGNB frequently contribute to nosocomial pneumonia and secondary bacteremia [18]. The infections caused by NFGNB have been commonly identified not only among immunocompromised or critically ill patients but also among immunocompetent hosts. Particularly, hospital-acquired infections and nosocomial outbreaks related to contaminated devices are reported. NFGNB infections are associated with high morbidity and mortality rates, highlighting the urgent need for effective therapeutic strategies [21].

### 2.1. Pathogenicity

Rare NFGNB pneumonia is typically opportunistic and favoured by healthcare exposure and host vulnerability. Key pathogenic features include persistence in moist hospital environments and biofilm formation on airways and devices (e.g., endotracheal tubes), which promotes persistence and tolerance to antibiotics. Given intrinsic and heterogeneous resistance patterns, accurate identification and MIC-guided therapy are crucial, particularly to distinguish colonization from true infection in ventilated or chronically diseased lungs [18].

In addition to classical virulence factors, several NFGNB species possess specialized secretion systems, such as the Type III and Type VI secretion systems (T3SS and T6SS), which facilitate the injection of effector proteins into host cells or competing bacteria. These systems are particularly well-characterized in *P. aeruginosa* and *Burkholderia* spp., where they contribute to immune evasion, interbacterial competition, and chronic colonization [22].

The epidemiology of rare NFGNB reveals a striking diversity, with certain species like *Burkholderia* and *Achromobacter* often identified as key players in chronic infections, particularly among patients suffering from CF. These organisms can silently inhabit the lung environment, contributing to persistent and challenging respiratory issues. Particularly, *B. cenocepacia* is considered one of the most virulent species among the *B. cepacia complex*, and its presence is a contraindication for lung transplants. Conversely, other species within this group, like *Alcaligenes* spp., are more frequently associated with opportunistic infections, posing significant risks to critically ill patients who are already vulnerable due to their weakened immune systems. Also, *Ochrobactrum* spp. infections typically occur in immunocompromised individuals or patients with tumours [3]. Similarly, *K. gyiorum* has been reported in chronic, non-healing infections, frequently in association with other microorganisms. Although its precise virulence factors are not fully understood, its recurrent isolation from sites of long-standing inflammation suggests a potential role in persistent infections, especially in patients with predisposing conditions [12].

In tertiary care hospitals, NFGNB can be found on many surfaces and instruments, including humidifiers used in ventilator machines, mattress surfaces, and various pieces of medical equipment. They can also be present on the skin of healthcare workers who come into close contact with patients. This raises concerns about horizontal transmission, as these resilient organisms can easily spread via contaminated fomites or through direct contact with caregivers’ hands, making infection control crucial in healthcare settings [6].

The accurate identification of these organisms is challenging due to their phenotypic similarities and the limitations of conventional biochemical methods. This is particularly true for *K. gyiorum*, which can be easily misidentified as other non-fermenting bacteria, such as *A. baumannii* complex or *Alcaligenes* spp., when relying solely on biochemical tests. The use of MALDI-TOF MS and 16S rRNA gene sequencing has been proven essential for the accurate detection of this rare pathogen in clinical specimens [12].

Indeed, NFGNB typically grow on standard culture media that show no haemolysis. Their growth is enhanced on chocolate agar, particularly for fastidious species such as *Burkholderia* spp. Several commercial systems have been developed to facilitate the identification and AST of clinically relevant microorganisms. Among these, API 20NE (bioMérieux, Marcy-l’Étoile, France) is a widely used manual system designed for the biochemical identification of NFGNB, offering a panel of 20 miniaturized tests interpreted via the APIWEB™ database. Automated platforms such as Phoenix (BD, Franklin Lakes, NJ, USA), MicroScan WalkAway (Beckman Coulter, Brea, CA, USA), and VITEK 2 (bioMérieux, Marcy-l’Étoile, France) provide integrated solutions for both identification and AST. The Phoenix system employs advanced nephelometry and a robust expert system to deliver rapid and reliable results. MicroScan WalkAway offers high-throughput capabilities and precise detection of emerging resistance patterns, supported by the LabPro software. VITEK 2, known for its speed and comprehensive database, delivers results within hours and integrates seamlessly with mass spectrometry systems for enhanced microbial profiling. These technologies have significantly improved laboratory workflows and diagnostic accuracy, although their performance may vary depending on the organism and clinical context. They have shown a strong capability in identifying NFGNB. However, it is worth noting that cases of misidentification do arise, and differentiating between the various species can present certain challenges. For example, these diagnostic tools may occasionally confuse *Burkholderia* spp. with *Achromobacter* spp. or *Ralstonia* spp. In such instances, it may be useful to consider additional testing to ensure accurate microbiological identification [3]. Comparative studies have demonstrated that MALDI-TOF MS significantly enhances the accuracy of species-level identification of NFGNB compared to conventional biochemical methods. For instance, Whistler et al. [22] reported a 30% reduction in misidentification rates when MALDI-TOF was used as a first-line diagnostic tool. Although MALDI-TOF MS has improved the routine identification of NFGNB, performance for rare taxa depends heavily on database completeness and local validation. When identification is ambiguous or limited to the genus level, or when the confidence level is low, confirmatory molecular methods (e.g., 16S rRNA gene sequencing) should be considered to avoid clinically relevant misidentification [22,23].

In respiratory specimens, MALDI-TOF MS should be used as the first-line method for rapid identification; however, results should always be interpreted in the clinical context, particularly to distinguish true infection from colonization. In case of low-confidence or genus-level identification, molecular confirmation (e.g., 16S rRNA gene sequencing) should be performed to minimise clinically relevant misidentification and support MIC-guided.

### 2.2. Drug Resistance

It is well documented that common NFGNB, including *P. aeruginosa*, *A. baumannii* complex, and *Stenotrophomonas* species, and rare ones, including *Achromobacter* spp., *Burkholderia* spp., *Sphingomonas* spp., *Alcaligenes* spp., *Brevimundimonas* spp., *Aeromonas* spp., *Roseomonas* spp., *Ochrobactrum* spp., and *K. gyiorum*, often present inherent resistance to multiple antibiotics [3,13].

Across rare NFGNB implicated in pneumonia, resistance is genus- and species-dependent and could be summarised into clinically meaningful profiles. Highly challenging taxa (e.g., *Achromobacter* spp., *Burkholderia cepacia* complex and *Pandoraea* spp.) often show a MDR profile with heterogeneous and unpredictable susceptibility, limiting the reliability of empiric regimens. Other genera exhibit characteristic intrinsic resistance patterns that can lead to inappropriate early therapy if unrecognised, whereas some taxa may retain activity to selected classes but remain variable across strains and therefore require MIC confirmation. A structured comparison of key resistance traits and commonly active agents across genera is provided in Supplementary Materials Table S1.

Conversely, *K. gyiorum* demonstrates a high degree of susceptibility to aminoglycosides, ciprofloxacin, imipenem, meropenem, and broad-spectrum cephalosporins. However, resistance to colistin and, in certain instances, to fluoroquinolones, has been documented. These findings underscore the significance of incorporating *K. gyiorum* into the differential diagnosis of chronic, non-healing infections and the utilisation of comprehensive microbiological investigations in such cases [12].

This complicates treatment options and poses significant challenges in clinical management. For instance, *Achromobacter* spp. exhibits intrinsic resistance to cephalosporins (with the exception of ceftazidime), aztreonam, ertapenem, and aminoglycosides. Among the characterised efflux pumps, AxyABM has been shown to play a significant role in the extrusion of cephalosporins, aztreonam, and chloramphenicol. Nevertheless, this is not the sole mechanism that contributes to antibiotic resistance. Indeed, *Achromobacter* spp. have been shown to possess the *bla*_OXA-114_ gene, which results in the constitutive production of the corresponding enzyme that hydrolyses piperacillin, ticarcillin, benzylpenicillin, and cephalothin [3].

*Alcaligenes faecalis* demonstrates resistance to multiple classes of antibiotics through various mechanisms. A primary factor is the production of β-lactamase enzymes, including carbapenemases, which enable the bacterium to degrade β-lactam antibiotics such as penicillins and cephalosporins. Additionally, efflux pumps reduce intracellular concentrations of antibiotics by actively expelling them. Furthermore, *A. faecalis* can form biofilms on medical devices and host tissues, further complicating its resistance profile [3].

*Burkholderia* species fall into two main groups: the *B. cepacia* complex (Bcc) and the *B. pseudomallei* complex (Bpc). Bcc exhibits intrinsic resistance to a wide range of antibiotics, including aminopenicillins, cephalosporins, aztreonam, ertapenem, ciprofloxacin, chloramphenicol, aminoglycosides, trimethoprim, fosfomycin, and colistin, which complicates treatment options for healthcare providers [24,25].

*Ochrobactrum* spp. are increasingly recognized for their intrinsic resistance to several classes of antibiotics. These organisms typically exhibit resistance to penicillins and most cephalosporins, and occasionally to carbapenems. The primary mechanism underlying this resistance is the chromosomally encoded *bla*_OCH_ gene, which produces an AmpC-like β-lactamase known as OCH. This enzyme hydrolyzes a broad spectrum of β-lactam antibiotics, contributing to therapeutic challenges in clinical settings. Additionally, *Ochrobactrum* spp. may harbour other resistance determinants, including efflux pumps and porin modifications, although these are less well characterized. Despite this resistance profile, *Ochrobactrum* spp. remains generally susceptible to ciprofloxacin and trimethoprim-sulfamethoxazole (TMP–SMX), which are considered effective empirical treatment options, particularly in immunocompromised patients or those with indwelling medical devices.

*K. gyiorum* is a rare NFGNB belonging to the *Alcaligenaceae* family, closely related to *Alcaligenes* spp., *Bordetella* spp., and *Achromobacter* spp. It was first identified in 2003 and has since been isolated from various chronic infections, including otitis media, urinary tract infections, and leg ulcers [12]. In a recent study, *K. gyiorum* was isolated for the first time from the sputum of two patients with neurodegenerative diseases (Alzheimer’s and Parkinson’s disease, respectively). Both strains were found to be resistant to fluoroquinolones, ciprofloxacin and levofloxacin, which may be related to the frequent use and abuse of different antibiotics [13]. *K. gyiorum* is often overlooked or misidentified because it resembles other bacteria and does not grow easily in standard laboratory conditions, requiring advanced tools like MALDI-TOF MS and 16S rRNA sequencing for accurate diagnosis [26]. The bacterium was susceptible to several antibiotics, including ciprofloxacin, ceftazidime, and gentamicin, but resistant to colistin [12].

The increasing prevalence of MDR NFGNB highlights the urgent need for novel therapeutic strategies, including the use of bacteriophage therapy, antimicrobial peptides, and synergistic antibiotic combinations. Ongoing research into the genomic and transcriptomic profiles of these organisms may also reveal new targets for antimicrobial development.

## 3. NFGNB Pulmonary Infections

### 3.1. Sphingomonas Pneumonia

The genus *Sphingomonas* (first proposed in 1989) comprises environmental NFGNB that have emerged as opportunistic causes of healthcare-associated infections, including pneumonia [27]. Clinically, *Sphingomonas* spp. pneumonia is most often linked to exposure to contaminated water systems and medical equipment (e.g., ventilator circuits, nebulizers, humidifiers) and occurs predominantly in patients with relevant comorbidities or healthcare exposure. Because these organisms may be infrequently encountered and can be misidentified by phenotypic methods, accurate species-level identification (typically by MALDI-TOF MS, with molecular confirmation when needed) is important to support appropriate antimicrobial selection and to distinguish true infection from colonization.

*S. paucimobilis* is commonly isolated from soil and various aquatic environments, including drinking water, sea water, river water, and mineral water. In healthcare settings, it is a well-recognized contaminant of hospital water systems, indwelling catheters, sterile intravenous fluids, hemodialysis devices, nebulizers, and ventilators. Its ability to form dense biofilms on pipes and filters facilitates its persistence and widespread dissemination within these environments. Their remarkable ability to survive and proliferate in low-nutrient conditions and to metabolize a wide array of carbon compounds contributes to their widespread environmental distribution [28,29,30,31,32].

Despite their pervasive presence, human infections caused by *Sphingomonas* spp. are relatively uncommon, often appearing as sporadic case reports or small series in the literature [28,33]. *S. paucimobilis* (formerly *Pseudomonas paucimobilis*) is the most frequently identified species in human clinical infections. These organisms are primarily recognized as opportunistic pathogens, causing infections predominantly in individuals with compromised immune systems or significant underlying comorbidities. However, cases of infection have also been described in immunocompetent subjects.

While *Sphingomonas* spp. has been implicated in a broad spectrum of infections, including bacteremia, meningitis, osteomyelitis, and urinary tract infections [34,35,36,37,38], lung infections and pneumonia represent a clinically significant manifestation, particularly in healthcare settings.

Transmission of *S. paucimobilis* often occurs through patient exposure to contaminated medical devices or solutions in the hospital environment. Nosocomial outbreaks have been directly attributed to contaminated liquids [39] and temperature sensors of ventilation equipment. Given *Sphingomonas* spp. documented ability to form dense biofilms and persist in hospital water systems, nebulizers, and ventilation equipment, its direct link to nosocomial outbreaks and ventilator-associated pneumonia (VAP) highlights a critical need for specific infection prevention strategies. The design, construction, and ongoing maintenance of hospital infrastructure, particularly water distribution systems and medical device reprocessing, are crucial infection control points. This extends beyond routine sterilization protocols to encompass engineering controls and water quality management strategies aimed at preventing the colonization and proliferation of *Sphingomonas* spp. within the hospital environment, thereby mitigating a persistent source of potential infection for vulnerable patients [40,41].

This organism causes a variety of community-acquired and nosocomial infections, usually occurring in immunocompromised hosts. In Taiwan, a retrospective study examined 55 cases of *S. paucimobilis* infections over a span of five years. Among them, 29 (52.7%) had community-acquired infections and 13 of them presented with primary bacteremia (44.8%). On the other hand, most of those who had health care-associated *S. paucimobilis* infections presented with pneumonia (10 of 26, 38.5%) and only 7.7% presented with catheter-related infection. The overall mortality rate was 5.5%. Diabetes mellitus was the most prominent risk factor for *S. paucimobilis* infections, followed by malignancy, chronic heart disease, and alcoholism [33]. Another study, also from Taiwan, concluded that the most common comorbidities identified in cases of *S. paucimobilis* bacteremia were malignancy, immunosuppressant use, and diabetes mellitus. Combining cases from that case series with those from the literature, the authors found a total of 42 patients with positive blood cultures for *S. paucimobilis*. Hospital-acquired bacteremia was identified in 69.0% of patients. Of the 42 patients, 25 (59.5%) had indwelling intravenous devices. The most common comorbidities identified were malignancy (57.1%), followed by immunosuppressant use (40.5%) and diabetes mellitus (11.9%). Primary *S. paucimobilis* bacteremia was found in 35.7% of patients. Catheter-related bloodstream infection was identified in 33.3% of patients, skin and soft tissue infection in 9.5%, pneumonia in 9.5%, urinary tract infection in 4.8%, biliary tract infection in 4.8%, and meningitis in 2.4% [28].

While the majority of reported *S. paucimobilis* infections are healthcare-associated (nosocomial), community-acquired cases are also documented, although they are sporadic. Community-acquired infections frequently present as primary bacteremia. Aspiration of oral bacteria, particularly following dental interventions or in the presence of dental abscesses, is an increasingly recognized pathway for pulmonary infection, even in individuals without overt immunosuppression [15]. This mechanism often leads to infections in the dependent pulmonary segments, most commonly the right lower lobe. Another explanation for *Sphingomonas* spp. infections lies in poor dentition or oral hygiene [42].

In 2022, Kumar et al. reported on a 59-year-old woman admitted for heart failure and pneumonia. Despite initial treatment, her condition worsened due to *S. paucimobilis* empyema, requiring surgical intervention and a change in antibiotics before she fully recovered [43]. This case, along with a review of three prior instances, highlights important patterns of this rare infection. First of all, aspiration is a common thread. Most cases, including one involving a transplant patient, another regarding an individual with alcoholism, and a third linked to an aspirated dental foreign body, suggest that aspiration (including inhaling foreign material into the lungs) is a primary cause. The localization of the infection in the right lower lung lobe further supports this interpretation. Furthermore, diabetes is a key risk factor. Two cases, including the most recent, involved patients with well-controlled diabetes, suggesting that even well-controlled diabetes can elevate the risk of infections and hospitalizations. Lastly, the four cases collectively point to risk factors for oral bacteria entering the lungs, such as advanced age, alcoholism, difficulty in clearing airways, and poor dental hygiene, as potential contributors to *S. paucimobilis* empyema [44,45,46].

In conclusion, *Sphingomonas* spp. infections are typically opportunistic and more often healthcare-associated, particularly in patients with comorbidities or device exposure. Early species-level identification and MIC-guided therapy are key to distinguishing infection from colonization and optimising treatment.

Nevertheless, clinically significant *Sphingomonas* spp. infections can also occur in seemingly immunocompetent individuals, although this appears uncommon. For example, Kar et al. described a 26-year-old immunocompetent patient with dengue haemorrhagic fever who developed secondary bacteremia due to *S. paucimobilis*, isolated from peripheral blood and bone marrow samples [36].

### 3.2. Aeromonas Pneumonia

*Aeromonas* spp. are Gram-negative, facultatively anaerobic bacteria widely distributed in aquatic environments like freshwater, river and estuarine water (brackish water), surface water, drinking water, polluted water bodies, and sewage sludge [47,48,49,50,51].

The primary route of infection is contact with contaminated stagnant fresh or brackish water in warm seasons, particularly during summer when bacterial counts peak [52,53]. It is increasingly recognized as an opportunistic human pathogen that can affect patients with chronic underlying conditions such as nephritis, diabetes, tumours, leukemia, hepatobiliary or pancreatic diseases, and patients with compromised immunity who are at higher risk of infection. Human infections are usually caused by *A. hydrophila*, *A. veronii* biovar *sobria*, and *A. caviae* [54]. These organisms can cause various infections, including gastroenteritis, wound infections, and septicemia, particularly in immunocompromised individuals. It can also cause respiratory tract infection, eye infection, osteomyelitis, meningitis, pelvic abscess, otitis, cystitis, endocarditis, peritonitis, cholecystitis, joint infection, necrotizing fasciitis, and folliculitis [55,56,57,58,59]. However, diarrhea is the most common clinical manifestation.

Recent genomic studies have revealed that the majority of clinical isolates belong to four species—*A. caviae*, *A. hydrophila*, *A. veronii*, and *A. dhakensis*—with *A. caviae* predominating in hepatobiliary infections and *A. hydrophila* more commonly associated with skin and soft tissue infections [60]. Their pathogenicity is attributed to a diverse arsenal of virulence factors, including cytotoxins, haemolysins, and the ability to form biofilms via quorum sensing mechanisms. Moreover, *Aeromonas* spp. exhibit intrinsic resistance to several antibiotics, such as penicillin and ampicillin, and harbor chromosomally encoded β-lactamase genes, complicating treatment strategies [60]. The emergence of colistin resistance genes, particularly *mcr-3-like* variants in *A. hydrophila* and *A. dhakensis*, further underscores the need for vigilant antimicrobial stewardship.

*Aeromonas* spp. are increasingly recognized as opportunistic pathogens that can cause a variety of extraintestinal infections, including pneumonia, particularly in immunocompromised individuals. Sakurai et al. [61] conducted a multicenter cohort study in Japan, identifying pneumonia as one of the less common but notable manifestations of *Aeromonas* infections (4.8% of cases). Although respiratory tract infections represent a minority of *Aeromonas* spp.-related clinical presentations, they are clinically significant due to their potential severity and diagnostic challenges. These infections may arise from aspiration of contaminated water; indeed, many cases have been reported after freshwater or saltwater near-drowning [62,63,64,65,66,67]. *A. hydrophila* was the most common *Aeromonas* spp. reported to cause pneumonia in drowning patients [65]. *Aeromonas* spp. pneumonia is possibly due to the aspiration of a large amount of water containing a high concentration of bacteria that can quickly lead to haemorrhagic pneumonia. On the other hand, these bacteria can cause a secondary hematogenous spread by entering the bloodstream through the peritoneal barrier reaching the thoracic tissue as well as pelvic tissue, lymph, gallbladder, and other areas. This mechanism of infection is generally associated with underlying comorbidities such as malignancy or chronic pulmonary disease and can be life-threatening [55]. In addition, predisposing factors for *Aeromonas* pneumonia involve alcohol and cigarette consumption [68].

*Aeromonas* spp. pneumonia is rare but can present with fulminant clinical courses, including in immunocompetent individuals. Epidemiological patterns suggest it should be actively considered after aspiration or near drowning in freshwater or brackish water, as well as in vulnerable hosts (e.g., immunosuppression or major comorbidities), given the potential for rapidly progressive and severe disease. Because susceptibility can vary across species and strains, early microbiological sampling (including blood cultures and respiratory specimens) and prompt reassessment of empiric therapy based on MIC-guided antimicrobial susceptibility testing are essential to minimise inappropriate early treatment. Representative published cases [63,69,70] are summarised in Table S2.

In addition to the more common community-acquired infections, rare cases of nosocomial pulmonary infection by *Aeromonas* spp. have also been reported. Srivastava et al. [71] described a rare case of VAP due to *A. hydrophila*. The patient, already on mechanical ventilation and renal replacement therapy, developed new-onset infection with elevated leukocyte counts. The isolate was MDR but responded to intravenous ciprofloxacin. The authors emphasize the importance of early detection and tailored antimicrobial therapy in ICU settings.

Besides *A. hydrophila*, *A. caviae* and *A. dhakensis* have also been implicated in rare but severe cases of pneumonia, often in immunocompromised patients or those with underlying conditions, and are also burdened by notable resistance profiles that complicate empirical treatment strategies. The reported mortality rate among patients with an extraintestinal infection caused by *A. dhakensis* ranges from 25.5% to 37.5%. This is considerably higher than the rate among those infected with other *Aeromonas* spp., which ranges from 0% to 14% [72,73]. Many similarities in the clinical features, predisposing factors and pathological manifestations exist between *A. dhakensis* pneumonia and *A. hydrophila* pneumonia. *A. dhakensis* is often misidentified as *A. hydrophila*, *A. veronii*, or *A. caviae* by commercial phenotypic tests in the clinical settings [74], which may lead to misdiagnosis and ineffective treatment. A correct identification of the pathogen may be of great importance for the successful treatment of the patients with *A. dhakensis* infection, and this may rely on molecular identification with the sequences of housekeeping genes [75].

Sha et al. reported a case of severe pneumonia caused by *A. dhakensis* in a 56-year-old male with a congenital atrial septal defect. The infection led to septic shock and required advanced interventions including extracorporeal membrane oxygenation (ECMO). Metagenomic next-generation sequencing (mNGS) confirmed the pathogen in both bronchoalveolar lavage fluid and blood samples. This study highlights the extreme virulence of *A. dhakensis*, which can cause rapid systemic deterioration and has a high sepsis-related mortality rate [76]. A fatal case of fulminant pneumonia and bacteremia caused by *A. dhakensis* occurred in a previously healthy 26-year-old man following a river swim; despite aggressive therapy, the patient died within hours. Notably, *A. dhakensis* was detected via both blood culture and mNGS underscoring the value of molecular diagnostics in rare aeromonad infections [77]. Luo et al. reported the case of a 26-year-old man with no history of lung diseases or other disorders who was admitted to the hospital with community-acquired pneumonia as the first symptom and developed serious conditions such as haemolytic uremic syndrome, multiple organ dysfunction, and haemorrhagic shock within a short period. He died after 13 h of admission, and the subsequent mNGS test confirmed the finally identified pathogen of infection as *A. dhakensis*. He had embarked on self-driving trips for 3 days before the onset of the disease. En route, he swam in a lake, which could have been the cause of *Aeromonas* spp. infection [78]. Moreover, Palanivel et al. described a rare case of community-acquired pneumonia complicated by acute respiratory distress syndrome (ARDS) caused by *A. jandaei* in an immunocompetent male, highlighting diagnostic and management challenges related to such rare, MDR pathogens [79]. Their intrinsic resistance to narrow-spectrum penicillins and variable susceptibility to β-lactams and colistin underscore the importance of species-level identification and susceptibility testing in guiding therapy [61].

### 3.3. Roseomonas Pneumonia

A new category of unnamed, pink-pigmented bacteria was identified in 1984 by Gilardi and Faur [80]. These non-fermenting organisms shared similar characteristics with *Methylobacterium extorquens* (formerly known as *Pseudomonas mesophilica*). The CDC later referred to this group of related organisms as the “pink coccoid” group [81].

In 1993, Rihs and colleagues formally established the bacterial genus *Roseomonas* based on DNA hybridization studies of 42 distinct strains of slow-growing, aerobic, pink-pigmented, Gram-negative bacteria [82]. The genus currently encompasses six species: *R. gilardii*, *R. cervicalis*, and *R. fauriae*, along with three other unnamed species (designated as genomospecies 4, 5, and 6). These bacteria have been found in aquatic environments as well as in a variety of clinical samples, including those from blood, wounds, urine, respiratory tract, peritoneal dialysis fluid, corneas, and bones [83,84,85].

As the genus *Roseomonas* was only recently established, there is limited clinical experience with infections caused by these bacteria. *Roseomonas* spp. generally do not pose a high risk to healthy individuals. However, in immunocompromised patients these bacteria can cause significant or even fatal disease. These infections are particularly concerning for people with conditions like leukemia, septicemia, and those undergoing cancer chemotherapy or dialysis [85,86].

Existing literature has indicated that bacteremia from the use of a central line is often the primary source of *Roseomonas* spp. infection [87]. Infections can also occur in other parts of the body, with the most common sites being the respiratory system, followed by wounds, bones, the peritoneum, the gut, and transplanted kidneys [86,88,89].

Nangarani et al. described the case of a *R. gilardii* associated empyema in 73-year-old man with COVID-19 with a history of HIV (well controlled by antiretroviral therapy), and diabetes [90]. Kaore et al. reported another case of community-acquired secondary bacterial lung infections caused by *Roseomonas* genomo-species in a patient with known pulmonary tuberculosis [91].

Diagnosis may be delayed because *Roseomonas* spp. often require prolonged incubation (frequently ≥48 h) and may be misidentified by conventional phenotypic methods. When available, MALDI-TOF MS can accelerate identification, while confirmatory molecular approaches (e.g., 16S rRNA sequencing) may be required in ambiguous cases, particularly for infrequently encountered species [92,93].

Therapeutic evidence remains limited, but available series and laboratory investigations indicate that antimicrobial susceptibility is variable and species dependent. Resistance to several β-lactams (including reduced activity of third-generation cephalosporins) has been reported, whereas higher activity is often observed for aminoglycosides and fluoroquinolones; carbapenem susceptibility has been described but is not uniform across datasets, reinforcing the need for MIC-guided treatment decisions [92,94]. In practice, when *Roseomonas* spp. is considered clinically significant (e.g., invasive infection or severe pneumonia in a high-risk host), empiric therapy should be promptly reassessed once species identification and susceptibility results are available, and source control (e.g., device management when relevant) should be considered [92,95].

### 3.4. Achromobacter Pneumonia

*A. xylosoxidans* was first described by Yabuuchi and Oyama in 1971 after isolation from ear secretions of patients with chronic otitis media and was included in the family *Alcaligenaceae* and more recently reinserted into the genus *Achromobacter* [96,97]. It is a Gram-negative, aerobic, motile, non-fermentative, catalase- and oxidase-positive bacillus, widely distributed in the aquatic environment (well water, tap water and swimming pools) but also in soil and plants [98]. In nosocomial environments it has been isolated from non-bacteriostatic saline solutions, dialysis solutions, computed tomography (CT) contrast solutions, ultrasound gel, and chlorhexidine gluconate solutions. It also colonizes mechanical ventilators, neonatal incubators, intravenous catheters, epidural catheters, and urinary catheters [99]. This organism has gradually been accepted as an emerging and opportunistic pathogen that can cause various nosocomial/non-nosocomial infections [100]. It is typically isolated in nosocomial infections affecting patients with hematologic or oncological disorders (30%), cardiac disease (21%), or immunosuppression (27%) [99] and relatively common among patients with CF, where the reported prevalence rate of colonization ranges from 5.3% to 13.1% [101,102]. In one study, *A. xylosoxidans* was found to be the most common NFGNB in patients with CF, second only to *P. aeruginosa* [99]. Chronic colonization with *A. xylosoxidans* in patients with CF has been associated with a decline in lung function and an increased risk of pulmonary exacerbation [103]: pulmonary infections by these germs are therefore quite related to the integrity of lung tissue, as in CF or bronchiectasis [102]. Innate MDR and a variety of virulence factors of *A. xylosoxidans* lead to the need for long-term antibiotic therapy, recalcitrance to treatment, association with poor clinical outcomes, and emergence as a problematic pathogen [104].

The innate resistance of *A. xylosoxidans* to aminoglycosides, aztreonam, tetracyclines, and certain penicillins and cephalosporins has been documented since the genus was first described in the 1970s. However, resistance mechanisms have only recently been described at the genomic level. Some of these genetic sequences share many similarities with those of other CF pathogens, such as *P. aeruginosa* and *B. pseudomallei*. The mechanism by which Achromobacteria can adhere, colonize, and subsequently infect the respiratory tract is unclear, but several shared and strain-specific virulence factors have been described:

- intrinsic factors of the genus that allow swimming motility via peritrichous flagella and can thus promote invasion of host cells.
- Cell membrane components such as LPS that, like other Gram-negative pathogens, induce key inflammatory cytokines, such as IL-6, IL-8, and TNF.
- Environmental advantage that allows survival in iron- and phosphorus-poor environments such as the human body, thanks to genes encoding high-affinity iron chelators and phosphate transporters.
- Denitrification similar to that of *P. aeruginosa*, allowing survival and proliferation in hypoxic and even anoxic environments [8].

Resistance patterns among *A. xylosoxidans* strains are not uniform, making treatment decisions more difficult. Intrinsic to most clinical isolates of *Achromobacter* spp. are Ambler class D β-lactamases, an Ambler class C β-lactamase, aminoglycoside-modifying enzymes, and RND-type multi-drug efflux pumps that make susceptibility to chloramphenicol, tetracyclines, and fluoroquinolones variable. Thus, *A. xylosoxidans* is characteristically resistant to all aminoglycosides and rifampicin, while it expresses variable resistance to TMP–SMX, ciprofloxacin and other quinolones. Most isolates are generally susceptible to carbapenems and anti-*Pseudomonas* penicillins [103]. Piperacillin-tazobactam, carbapenems, ceftazidime or TMP–SMX should be considered as first-line empiric treatment options while minocycline is another alternative therapeutic option with in vitro activity against *Achromobacter* spp. The new β-lactam/β-lactamase inhibitors are not active in vitro and have not been studied clinically [103]. In one study [5], high values of inhibition zone diameter were observed with cefiderocol among the included isolates. This may highlight the potential of cefiderocol as a promising treatment for serious *Achromobacter* spp. infections, although clinical evidence on in vivo activity is still limited.

### 3.5. Burkholderia Pneumonia

The genus *Burkholderia* includes more than one hundred species, but from a clinical standpoint, it is traditionally divided into two medically relevant complexes. The first is the *B. cepacia* complex (Bcc), composed of more than twenty genetically distinct yet closely related non–lactose-fermenting Gram-negative species [105]. The second is the *B. pseudomallei* complex, which comprises *B. pseudomallei*, the causative agent of melioidosis, and *B. mallei*, responsible for glanders, both associated with severe infections in tropical and subtropical regions and requiring prolonged antimicrobial therapy [106,107].

Bcc organisms are widely distributed in soil and water and exhibit remarkable environmental persistence, surviving in nutrient-poor or antiseptic-containing solutions—including disinfectants, chlorhexidine, intravenous fluids, nebulizer solutions, and ventilator tubing—contributing to repeated nosocomial clusters linked to contaminated medical products [108]. Clinically, the greatest impact of Bcc occurs in CF (Table 1), where colonization remains uncommon but significant, with prevalence typically ranging from two to four percent in major international registries [109]. Among CF isolates, *B. multivorans* is most frequent, whereas *B. cenocepacia* is regarded as the most virulent due to enhanced transmissibility, epidemic spread in care centers, and association with worse outcomes; the fulminant “cepacia syndrome” remains the most feared presentation, with necrotizing pneumonia, bacteremia, and high short-term mortality [110].

Beyond CF, Bcc infections are increasingly recognized in non-CF bronchiectasis, advanced chronic obstructive pulmonary disease (COPD), hematologic malignancies, solid-organ transplant recipients, and critically ill patients exposed to invasive devices and broad-spectrum antibiotics [111]. Historically, Bcc colonization was considered a relative or absolute contraindication to lung transplantation due to excess post-transplant mortality, particularly among recipients infected with epidemic *B. cenocepacia* lineages [111,112]. More recent analyses indicate that outcomes are species- and lineage-dependent: patients infected with *B. multivorans* and those harboring sporadic, non-epidemic *B. cenocepacia* strains may achieve post-transplant survival comparable to uninfected CF recipients when managed in experienced centers using tailored perioperative strategies [112].

The Bcc displays both intrinsic and acquired AMR mechanisms. Intrinsic resistance is driven by the low permeability of the outer membrane, multiple chromosomally encoded β-lactamases—most notably the class A PenA enzyme and inducible AmpC-like variants—and highly conserved RND (Resistance–Nodulation–Division) efflux pumps that actively extrude several antibiotic classes. Biofilm formation and quorum-sensing-regulated adaptive responses further enhance tolerance and persistence, particularly in the chronically inflamed airways of CF patients [113,114]. Acquired resistance has also been documented, including plasmid-mediated carbapenemases such as *bla*_NDM-1_ and *bla*_NDM-5_, recently identified in respiratory isolates and contaminated ventilator tubing during outbreaks of VAP [115].

Clinically, these features translate into high baseline MICs and heterogeneous susceptibility profiles, limiting the reliability of standard empiric β-lactams and contributing to treatment failure or persistence (especially in biofilm-associated disease) [116]. Accordingly, early species/strain-level identification and MIC-guided optimisation are essential, and severe infections may require combination therapy and source control when device- or product-associated acquisition is suspected [117].

Clinical manifestations of Bcc are heterogeneous and influenced by the underlying host condition. In CF patients, chronic colonization is strongly associated with an accelerated decline in lung function, an increased frequency of pulmonary exacerbations, and a poorer prognosis compared with colonization by other NFGNB [118]. Acute pulmonary exacerbations typically present with fever, dyspnea, purulent sputum, and new infiltrates on chest imaging, while “cepacia syndrome” represents a dramatic evolution, with rapid onset of necrotizing pneumonia, bacteremia, and systemic inflammatory response syndrome, often progressing to multi-organ failure [110,119].

Although rare and only sporadically described, Bcc infection has been reported in patients with advanced COPD and bronchiectasis, presenting as an acute pulmonary exacerbation with productive cough, dyspnea and bronchiectatic changes on CT imaging [120,121]. In immunocompromised hosts, including oncology and haemato-oncology patients, as well as critically ill individuals in adult and neonatal ICUs, Bcc can cause severe hospital-acquired infections such as bacteremia and VAP, frequently linked to contaminated medical products, intravenous fluids or devices [118]. In solid-organ transplant recipients, particularly those undergoing lung transplantation, *B. cepacia* complex has been associated with severe post-transplant pneumonia and poor clinical outcome [111]. Radiologically, findings range from peribronchial thickening and mucus plugging to tree-in-bud nodules, lobar or segmental consolidation, and, in more severe presentations, cavitary lesions. Laboratory findings are nonspecific but include elevated inflammatory markers such as C-reactive protein (CRP) and procalcitonin, and variable leukocyte responses. Although the available evidence is limited, reported cases of “cepacia syndrome” almost exclusively involve patients with CF chronically colonized by *B. cenocepacia*, often for several years before onset. No consistent host-related clinical risk factors have been identified, apart from the underlying CF disease and the predominance of highly virulent strains such as ET12 [110]. Although classically linked to CF, *B. cepacia* complex infection has also been documented in patients without CF, including those with advanced COPD [122].

TMP–SMX remains the most reliable traditional agent against Bcc, yet resistance is already 30.3%, and the species carries multiple intrinsic resistance mechanisms which severely limit the activity of many antibiotics [123]. Accordingly, real-world cohorts describe high resistance to commonly used agents (including piperacillin–tazobactam, ceftazidime, levofloxacin and carbapenems), while TMP–SMX remains comparatively more reliable, albeit with non-negligible resistance; key resistance rates of the commonly used antimicrobials are summarized in Table S3.

Because of this extensive resistance, interest has shifted toward new β-lactam/β-lactamase inhibitor combinations. Among them, ceftazidime–avibactam (CZA) shows the most consistent in vitro performance: in a systematic review of nine studies, avibactam reduced ceftazidime MICs by 4–32 fold, with 67–97% of Bcc isolates susceptible and MIC_90_ values generally between 8 and 16 mg/L [124]. Other emerging combinations—such as meropenem–vaborbactam, cefepime–taniborbactam, and piperacillin–avibactam—have shown activity in selected in vitro studies, though clinical evidence remains scarce. Clinically, these newer options should be positioned mainly as MIC-guided salvage/adjunctive therapies for severe infections and MDR isolates, often within combination regimens when first-line active agents are unavailable or have failed.

Cefiderocol represents a therapeutic option for multidrug-resistant *Burkholderia* infections, showing low MIC values (MIC90 0.5 μg/mL) and reliable in vitro activity against *Burkholderia* spp. and the Bcc [125]. A recent case report in CF described marked clinical improvement and a 5-month exacerbation-free period following cefiderocol therapy for chronic MDR Bcc infection, despite persistent colonization [126].

Finally, interest in adjunctive bacteriophage therapy has grown in recent years, with several case reports describing clinical stabilization and improved respiratory function in CF patients treated with personalized phage cocktails targeting MDR Bcc [127]. Although still experimental, this approach represents one of the few innovative therapeutic avenues for patients with otherwise untreatable infections.

The Bcc continues to represent a formidable challenge in respiratory medicine. Its ability to colonize and persist in the airways, its intrinsic and acquired resistance mechanisms, and its association with severe clinical syndromes such as “cepacia syndrome” make it one of the most difficult NFGNB to manage. Treatment requires careful microbiological characterization, judicious use of combination regimens, and consideration of novel agents such as CZA, aztreonam–avibactam, and cefiderocol. For transplant candidates, decisions must be guided by species- and strain-level data rather than by categorical exclusion. Looking forward, the integration of innovative strategies such as bacteriophage therapy may expand the therapeutic armamentarium, although robust clinical evidence is still needed.

### 3.6. Pandoraea Pneumonia

*Pandoraea* species are members of the family Burkholderiaceae; they are aerobic, motile, non-spore-forming, NFGNB. Clinically relevant species include *P. apista*, *P. pulmonicola*, *P. pnomenusa*, *P. sputorum*, and *P. norimbergensis* [128]. They are considered emerging NFGNB increasingly recognized as opportunistic pathogens, particularly in patients with CF, structural lung disease, and immunocompromised states. Historically underdiagnosed due to phenotypic misidentification as Bcc, improved molecular diagnostics have clarified their taxonomic status and clinical relevance [129,130]. MALDI-TOF MS improves identification when updated databases are available. However, underdiagnosis likely persists in settings without molecular confirmation. Clinical presentations range from chronic airway colonization, especially in patients with chronic lung disease such as those affected by CF, to invasive infections like VAP and catheter-related bacteremia, that may occur in solid organ transplant recipients, in patients with hematologic malignancies and critically ill ICU patients. Respiratory colonization in CF represents the most frequently reported context. A recent systematic review [131] confirmed respiratory involvement as predominant, though clinical impact varies from benign colonization to recurrent exacerbations. Clinical outcomes range from stable colonization to lung function decline. A defining feature of *Pandoraea* spp. is multidrug resistance, with heterogeneous and unpredictable susceptibility patterns. Genomic studies reveal efflux pumps, permeability changes, beta-lactamase production and quorum sensing systems [132] as major mediators of antimicrobial resistance, though virulence mechanisms remain incompletely defined. Biofilm production likely contributes to chronic airway persistence and device colonization. Resistance to penicillins and cephalosporins is common; carbapenem susceptibility is variable. High-level resistance frequently limits therapeutic options. Activity of Fluoroquinolones and TMP-SMX is heterogeneous and isolate-dependent [132]. Currently a standardized treatment algorithm is not available. Principles of therapeutic strategy require that accurate microbiological tests and phenotypic sensitivity tests be mandatory. Management remains individualized and susceptibility-driven. In severe infections, combination therapy should be considered and infected devices should be removed as soon as possible when applicable. Emerging agents such as novel beta-lactam/beta-lactamase inhibitor combinations may have theoretical utility, though evidence is limited [3,132]. Outcomes depend primarily on host factors and adequacy of antimicrobial therapy. Data remain limited, and prospective studies are needed [3].

### 3.7. Elizabethkingia Pneumonia

*Elizabethkingia* spp. are Gram-negative, non-motile, aerobic, non-fermenting bacilli. Eight species currently comprise the genus *Elizabethkingia*: *E. meningoseptica*, *E. miricola*, *E. anophelis*, *E. bruuniana*, *E. ursingii*, *E. occulta*, *E. argenteiflava*, and *E. umeracha*. *Elizabethkingia* spp. are emerging pathogens implicated in healthcare-associated infections. Of all members of the genus *Elizabethkingia*, *E. anophelis* and *E. meningoseptica* are the predominant species as nosocomial pathogens and play a growing role in nosocomial outbreaks [133,134,135]. While *Elizabethkingia* spp. rarely cause disease in healthy individuals, they have been shown to cause infections in newborns, elderly, and immunocompromised individuals, often manifesting as pneumonia [136,137]—mostly ventilator-associated—bacteremia, meningitis (where *E. meningoseptica* is prevalent), urinary tract infections, skin and soft tissue infections. Main risk factors for acquiring infections by *Elizabethkingia* spp. are admission to an ICU, invasive devices and procedures such as need for mechanical ventilation, central venous catheters and hemodialysis, prolonged hospital stay, prior use of multiple broad-spectrum antibiotics, immunocompromised status (e.g., cancer, diabetes), chronic and severe illnesses [133,134]. These bacteria are often found in water sources and moist hospital environments, such as sink basins, taps, and ventilator tubing, which can lead to nosocomial (hospital-acquired) outbreaks. The prevalence of infections caused by *Elizabethkingia* spp. has recently increased in several countries. Although still rare, invasive *Elizabethkingia* spp. infections have high mortality rates, ranging from 20% to 45%. To date, there are no definitive literature data suggesting clearly defined therapeutic strategies for the management of infections caused by *Elizabethkingia* spp. These microorganisms show intrinsic resistance to β-lactams, carbapenems, and polymyxins. The choice of the correct antimicrobial therapy for the treatment of infections caused by *Elizabethkingia* spp. must be guided by careful antimicrobial sensitivity testing and, especially in cases of severe infections, must include combination treatment [138] with one of the following: minocycline/doxycycline, moxifloxacin/levofloxacin, rifampicin, piperacillin-tazobactam, TMP-SMX. Literature data suggest a possible role for combination therapy with vancomycin. Given the potential ability to form biofilms, it is suggested that combination treatment against this microorganism should include the use of molecules potentially active on the biofilm [139].

### 3.8. Kerstersia gyiorum Pneumonia

*Kerstersia gyiorum* is a rare, opportunistic, NFGNB bacterium that belongs to the genus *Kerstersia* in the family *Alcaligenaceae*, which includes *K. gyiorum* and *K. similis*. This genus was originally classified within the family *Alcaligenaceae* alongside *Alcaligenes* spp., *Achromobacter* spp., *Bordetella* spp., and *Pigmentiphaga* spp. The specific epithet ‘gyiorum’ (named after the Greek word for “from the limbs” as this species was primarily isolated from lower-extremity wounds) was attributed due to isolation source often from limb wound swabs [140]. It has been reported as causative agent of respiratory tract infections [13,141,142], chronic ear infection, chronic lower limb infection, hemorrhagic dermatitis, chronic venous insufficiency, urinary tract infection, thromboangiitis obliterans, and chronic osteomyelitis [141]. The previous paucity of reported cases of human *Kerstersia* spp. infections have increased recently due to the possibility of accessing molecular diagnostics, particularly 16S rRNA gene sequencing and MALDI-TOF MS, in the identification of this microorganism. Literature data suggest that risk factors for *K. gyiorum* infection include immunocompromised status, previous and repeated hospitalizations, mechanical ventilator use, and surgical procedures. These bacteria have shown resistance to aminoglycosides (gentamicin, amikacin), azithromycin, and some cephalosporins; moreover, resistance to ciprofloxacin and TMP-SMX has been reported in some strains. Treatment typically involves targeted antibiotic therapy that should be guided by accurate antimicrobial susceptibility testing. The antibiotics used for treatment of patients with severe infection varied in previous reports and often comprised piperacillin/tazobactam, cefepime, ceftazidime, carbapenems (imipenem or meropenem) [141,143]. Treatment may require long-term monitoring due to its tendency to cause chronic, relapsing infections.

NFGNB: non-fermenting Gram-negative bacteria; ICU: intensive care unit; MDR: multi-drug resistant; BAL: bronchoalveolar lavage; MALDI-TOF MS: matrix-assisted laser desorption/ionization time-of-flight mass spectrometry; ID: identification; TMP-SMX: trimethoprim-sulphamethoxazole; MIC: minimum inhibitory concentration; FQ: fluoroquinolones.

## 4. Treatment of NFGNB Pneumonia

**Figure 1:**
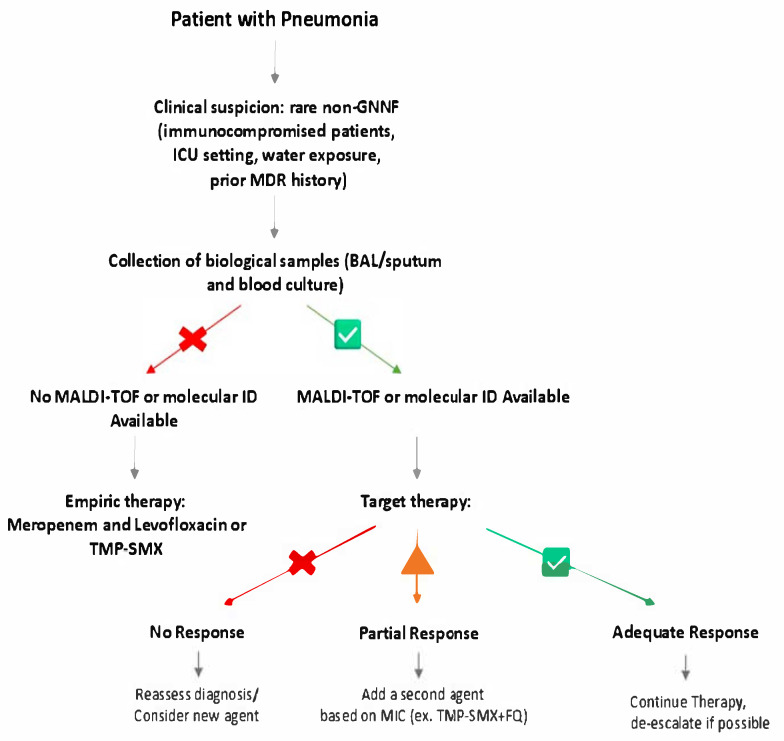
Therapeutic algorithm.

As a general rule, when a NFGNB pneumonia is suspected, empiric therapy should be broad (e.g., antipseudomonal carbapenem + fluoroquinolone or TMP-SMX) until speciation and susceptibilities tests are available (Figure 1). Definitive treatment of infections caused by NFGNB must be based on phenotypic susceptibility testing. Given their multiple capacities to express resistance factors, which are not easily explored with automated diagnostic systems, treatment cannot remain confined to an empirical approach since it may fail due to intrinsic resistance. Treatment must be supported by precise information from microbiology laboratories, which, on a case-by-case basis, can also integrate, where deemed necessary, the phenotypic susceptibility testing obtained with automated methods, also under the guidance of trained clinicians. Carbapenems, fluoroquinolones, TMP-SMX and minocycline are often active, depending on the species. In the case of severe infections caused by MDR strains, the use of combination therapies may also be considered, and new treatment options may arise from drugs that are going to be introduced soon.

Each individual species of rare NFGNB exhibits specific characteristics of potential antimicrobial resistance. However, even within the same species, there can be wide variability in antimicrobial susceptibility. For this reason, each individual microorganism of clinical interest must necessarily be carefully characterized by phenotypic susceptibility testing by the microbiology laboratory [3,144,145]. Once the organism is identified and susceptibilities available, narrow therapy to the most active, least toxic agent should be adopted, monitoring clinical response and adjusting therapy accordingly. The absence of clinical response may indicate resistance, mixed infection, or the need for adjunctive measures. It is also always important to correlate microbiology with clinical picture and to establish whether it is just colonization or a true infection. For the above reasons, consultation with infectious diseases specialists is recommended. The suggested antimicrobial treatments for pneumonia caused by NFGNB are summarized below and in Table 2.

- *Achromobacter* spp., mostly *A. xylosoxidans*, show intrinsic resistance to cephalosporins (except ceftazidime), aztreonam, aminoglycosides, and ertapenem. They could express the presence of efflux pumps, possible metallo-β-lactamases (e.g., VIM, IMP), and OXA-114-like chromosomal genes. Literature data report some success with carbapenems (meropenem, imipenem), ceftazidime, cefoperazone/sulbactam, piperacillin-tazobactam, ticarcillin, TMP-SMX, minocycline, eravacycline, levofloxacin, ciprofloxacin. If resistant to carbapenems, ceftazidime/avibactam + piperacillin-tazobactam and cefidercol could represent a new option. In severe infections combination treatment (ceftazidime/meropenem/imipenem-cilastatin/cefiderocol + TMP-SMX) is suggested. Therapy failures are common if empirical regimens are not active. Delayed effective therapy is associated with worse outcomes.
- The *Burkholderia cepacia* complex is intrinsically resistant to most β-lactams (generally with the possible exceptions of ceftazidime, piperacillin/tazobactam, and meropenem), aminoglycosides, and polymyxins. Furthermore, it can acquire resistance to other classes of antibiotics through various mechanisms, such as drug-modifying enzymes, reduced membrane permeability, modification of the antimicrobial target, production of efflux pumps, and reduced outer membrane permeability.

For this reason, treatment of these microorganisms, after adequate and complete phenotypic testing by the microbiology laboratory, involves the use of the following antimicrobial agents, including a possible combination: ceftazidime, TMP-SMX, levofloxacin, minocycline, and meropenem. If phenotypic testing reveals carbapenem resistance, the following treatment options may be evaluated: CZA or cefiderocol. If resistance to carbapenems and CZA is confirmed, the combination of CZA + piperacillin-tazobactam, or imipenem/relebactam or cefiderocol is proposed. Future options include the use of combinations with new β-lactams inhibitors, such as cefepime/zidebactam.

- Regarding *Ochrobactrum* spp., where *O. anthropi* is the most often cited species in human infections, often in immunocompromised patients, they are generally resistant to many β-lactams. Carbapenems, fluoroquinolones, and TMP-SMX could be active but therapy must be guided by tested susceptibilities.
- *Aeromonas* spp. are typically susceptible to fluoroquinolones, third-generation cephalosporins, carbapenems, and TMP-SMX; they are usually resistant to penicillins. In severe diseases, a combination including a carbapenem or extended-spectrum cephalosporin plus a fluoroquinolone may be considered.
- Regarding *Roseomonas* spp., especially *R. gilardii*, most often involved in human diseases (bloodstream infections, local and pulmonary infection), limited data suggest susceptibility to fluoroquinolones and possibly carbapenems, but treatment should be guided by MIC results.
- *Elisabethkingia* spp. (e.g., *E. meningoseptica*, *E. anophelis*) are emerging nosocomial pathogens with frequent multidrug resistance. They are intrinsically resistant to most β-lactams, carbapenems and polymyxin and could present many antimicrobial resistance mechanisms such as production of metallo-β-lactamases (BlaB, GOB). In vitro, isolates typically could show high susceptibility to minocycline, TMP-SMX, and rifampicin. Susceptibility to fluoroquinolones may be variable; tigecycline may have limited activity. Possible therapeutic options include combination therapies that may combine the following: minocycline/doxycycline, moxifloxacin/levofloxacin, rifampicin, piperacillin-tazobactam, TMP-SMX. A possible role for combination therapy with vancomycin is currently being hypothesized.
- *Chryseobacterium indologenes* and related species have been described in nosocomial pneumonia, particularly in lungs of immunocompromised or ventilated patients. They are often resistant to many β-lactams and carbapenems, but may be susceptible to quinolones, TMP-SMX, piperacillin/tazobctam, tigecycline or newer agents but therapy should follow susceptibility testing. Rifampin could be considered as a part of treatment.
- For *Alcaligenes* spp., most often *A. faecalis* occasionally reported in respiratory infections, generally in hospital settings, susceptibility is variable; carbapenems except ertapenem and fluoroquinolones may be effective, and in some cases also piperacillin-tazobactam and tigecycline, but therapy should be guided by susceptibility tests. In severe diseases, combination treatment is suggested (carbapenems + tigecycline/aminoglycosides/fluoroquinolones).
- *Ralstonia* spp. (most often *R. pickettii*) are intrinsically resistant to colistin and may express variable susceptibility to ceftazidime, cefepime, carbapenems, aminoglycosides. Therapeutic options could be TMP-SMX, fluoroquinolones, and piperacillin-tazobactam, but treatment should be guided by susceptibility tests and combination therapy may be needed in severe cases.
- For *Cupriavidus* spp. (most often *C. pauculus*) limited data exist regarding susceptibility profile; therapy must rely on MIC data.
- *Sphingomonas* spp. (most often *S. paucimobilis*) tend to be susceptible to carbapenems, fluoroquinolones, TMP-SMX, and tetracyclines, but therapy should be tailored according to susceptibilities.
- *Rhizobium* spp., *Empedobacter* spp., *Brevundimonas* spp. are genera very rarely implicated in human pulmonary disease; no standardized treatment guidances are reliable and therapeutic decisions must be individualized based on susceptibility tests. For *Brevimundimonas* spp., cephalosporins, penicillins and aminoglycosides have been suggested as potentially active but treatment decisions should be confirmed by susceptibilty tests.
- *K. gyiorum* treatment should be guided by accurate antimicrobial susceptibility testing. The antibiotics suggested for treatment of patients with severe infection are piperacillin/tazobactam, cefepime, ceftazidime, carbapenems (imipenem or meropenem). Treatment may require long-term monitoring due to its tendency to cause chronic, relapsing infections.
- *Pandoraea* spp. treatment should be individualized and susceptibility-driven due to the antimicrobial resistance related to this microorganism. Resistance to penicillins and cephalosporins is common and carbapenem susceptibility is variable. High-level resistance frequently limits therapeutic options. Activity of fluoroquinolones and TMP-SMX is heterogeneous and isolate-dependent. Currently, a standardized treatment algorithm is not available. In severe infections, combination with active agents like imipenem-cilastatin, fluoroquinolones, TMP-SMX, aminoglycosides are suggested. Emerging agents such as novel β-lactam/β-lactamase inhibitor combinations may have theoretical utility, though evidence is limited.

## 5. Conclusions

Rare NFGNB should be considered in immunocompromised, critically ill, ventilated, or hospital exposed patients, particularly when conventional pathogens are not recovered. Because resistance patterns among NFGNB vary widely, early species level identification—through MALDI-TOF MS, 16S rRNA sequencing, or equivalent methods—is essential. Empiric therapy is frequently inadequate due to intrinsic resistance; therefore, management must be guided by MIC values and susceptibility testing, with a formal reassessment of clinical response within 48–72 h to optimize or adjust therapy.

Among available treatments, TMP-SMX, fluoroquinolones, minocycline, rifampin, and carbapenems often remain the most reliable options, depending on genus-specific susceptibility profiles. Newer agents such as cefiderocol and novel βlactam/βlactamase inhibitor combinations (e.g., ceftazidime/avibactam, cefepime/zidebactam) show promise against MDR isolates, but data in these rare genera remain extremely limited [146,147]. Given the high risk of therapeutic failure or relapse—especially when treating poorly characterized organisms—combination therapy is recommended in severe or refractory infections, and empirical monotherapy should be avoided in immunocompromised or critically ill patients.

Close collaboration with infectious disease specialists and antimicrobial stewardship teams is strongly advised to ensure appropriate antibiotic choices and dosing strategies. Major obstacles in the management of pneumonia due to these rare organisms include the scarcity of prospective data, heterogeneity of published case reports, incomplete MIC reporting, and limited long-term follow-up. Moreover, in vitro susceptibility does not always correlate with in vivo efficacy, particularly in pneumonia, biofilm-associated infections, and hosts with impaired immunity. Persistent diagnostic uncertainty—especially distinguishing colonization from true infection—remains a significant clinical challenge.

## Figures and Tables

**Table 1:** *Burkholderia cepacia* complex in respiratory disease: clinical settings, features and treatment considerations.

Clinical Setting/Risk Profile	Typical Manifestations (Clinical, Radiological)	Complications
Cystic fibrosis (CF)	Chronic colonization → accelerated lung-function decline, frequent pulmonary exacerbations. Acute exacerbations: fever, dyspnea, purulent sputum, new infiltrates. Radiology: peribronchial thickening, mucus plugging, tree-in-bud nodules, lobar/segmental consolidation; in severe cases cavitations.	**Cepacia syndrome:** necrotizing pneumonia, bacteremia, SIRS, multi-organ failure. Rapid clinical deterioration, high short-term mortality (especially with *B. cenocepacia*/ET12).
Non-CF bronchiectasis	Exacerbation with cough, dyspnea, purulent sputum. CT: bronchiectasis, mucus plugging.	Progression to severe infection; potential chronic colonization.
Advanced COPD (non-CF)	Acute exacerbation with dyspnea, productive cough; radiological bronchiectatic changes.	Risk of severe pneumonia in presence of comorbidities.
Immunocompromised hosts (hematologic malignancies, oncology, transplant, neonatal/adult ICU)	Hospital-acquired infections: bacteremia, VAP. Often linked to contaminated medical products, fluids, ventilator tubing.	Severe sepsis, multi-organ failure. High mortality, especially post-transplant.
Lung transplantation candidates/recipients	Severe pneumonia; post-transplant decompensation.	Historically poor outcomes, especially with epidemic *B. cenocepacia*. Outcomes now species/lineage-dependent.

COPD: chronic obstructive pulmonary disease; ICU: intensive care unit; VAP: ventilator-associated pneumonia; CT: computed tomography; SIRS: systemic inflammatory response syndrome.

**Table 2:** Treatment of infections caused by non-fermenting Gram-negative bacilli (NFGNB).

Pathogen	Active Agents	Key Notes
*Burkholderia cepacia complex*	Ceftazidime; TMP-SMX; Levofloxacin; Minocycline/Doxycycline; Meropenem. If resistant: CZA; Cefiderocol.	Severe disease: combination therapy. Options: CZA + Piperacillin–tazobactam; Cefiderocol ± Imipenem-relebactam/meropenem-varbobactam. Future option: Cefepime–zidebactam.
*Achromobacter xylosoxidans*	Carbapenems (not ETP); Ceftazidime; FQs; Minocycline; Eravacycline; TMP-SMX. If resistant: CZA; Cefiderocol.	Intrinsic resistance: most cephalosporins, aztreonam, aminoglycosides, ertapenem. Severe disease: combination therapy (e.g., ceftazidime/meropenem/imipenem-cilastatine + TMP-SMX).
*Elizabethkingia* spp.	Minocycline/Doxycycline; FQs; Rifampicin; Piperacillin–tazobactam; TMP-SMX; Cefiderocol.	Intrinsic resistance to β-lactams, carbapenems, polymyxins. Severe disease: Minocycline + Rifampicin.
*Alcaligenes* spp.	Carbapenems (not ertapenem); FQs; Piperacillin–tazobactam; Tigecycline.	Severe disease: Carbapenem + Tigecycline/Aminoglycosides/FQ.
*Ochrobactrum* spp.	FQs; TMP-SMX.	Resistant to most β-lactams; variable carbapenem susceptibility.
*Ralstonia* spp.	TMP-SMX; FQs; Cefotaxime; Aminoglycosides; Tigecycline.	Variable: Ceftazidime, Cefepime, carbapenems, Aminoglycosides. Intrinsic resistance: Colistin.
*Chryseobacterium* spp.	TMP-SMX; FQs; Piperacillin–tazobactam; Tigecycline.	Consider adding Rifampin.
*Brevundimonas* spp.	Cephalosporins; Penicillins; Aminoglycosides.	Susceptibility often broad but variable.
*Sphingomonas* spp.	FQs; TMP-SMX; Tetracyclines.	Carbapenems may be active.
*Kerstersia gyiorum*	Piperacillin–tazobactam; Cefepime; Ceftazidime; Carbapenems.	Very limited data → based on MIC.
*Aeromonas* spp.	FQs; 3rd gen cephalosporins; Carbapenems; TMP-SMX.	Intrinsic resistance: Penicillins.
*Pandoraea* spp.	Imipenem–cilastatin; FQs; TMP-SMX; Aminoglycosides (in combination).	Resistant to most β-lactams.

TMP-SMX = Trimethoprim–sulfamethoxazole; FQs = Fluoroquinolones; CZA = Ceftazidime–avibactam.

## Data Availability

No new data were created or analyzed in this study. Data sharing is not applicable to this article.

## Supplementary Materials

The following supporting information can be downloaded at: https://www.mdpi.com/article/10.3390/antibiotics15050465/s1. Table S1: Comparative overview of antimicrobial resistance and susceptibility patterns among common and rare Non-Fermenting Gram-Negative Bacilli (NFGNB), Table S2: Representative cases of A. hydrophila pulmonary infections. Table S3: Resistance rates of Burkholderia cepacia complex (Bcc) to commonly used antimicrobial agents.
