# Supplementary material for: Managing Pneumonia Due to Rare Non-Fermenting Gram-Negative Bacteria: Epidemiology, Risk Factors and Therapeutic Strategies
Source: Antibiotics (Basel). 2026 May 4;15(5):465. doi: 10.3390/antibiotics15050465 (PMC13203845; doi:10.3390/antibiotics15050465)
Supplement: Supplementary file 1 [file antibiotics-15-00465-s001.zip › antibiotics-4228961-supplementary.pdf]

Table S1. Comparative overview of antimicrobial resistance and susceptibility patterns among common and rare Non-Fermenting Gram-Negative Bacilli (NFGNB).

Pathogen category	Genus/Species	Typical resistance Profile	Notable Susceptibilities
**Common NFGNB**	*P. aeruginosa, A. baumannii* complex, *Stenotrophomonas* spp.	High inherent resistance to multiple classes (e.g., penicillins, 1st/2nd gen. cephalosporins)	Variable; *Stenotrophomonas* is notably susceptible to TMP-SMX[1].
**Rare NFGNB**	*Achromobacter* spp., *Burkholderia* spp., *Alcaligenes* spp., *Brevimundimonas* spp., *Aeromonas* spp., *Roseomonas* spp., *Ochrobactrum* spp.	Broad intrinsic resistance (efflux pumps, β-lactamases). Often resistant to aminoglycosides and polymyxins.	Highly variable; requires specific susceptibility testing.
**Focus Species**	*Kerstersia gyiorum*	Colistin, occasionally fluoroquinolones.	Aminoglycosides, carbapenems, ciprofloxacin, broad-spectrum cephalosporins.

[1]TMP-SMX: Trimethoprim-sulfamethoxazole.

Table S2. Representative cases of *A. hydrophila* pulmonary infections.

Author [ref.]	Patient age/sex	Underlying conditions	Clinical presentation	Therapeutic Support	Outcome
**Nagata *et al.* [69]**	75/F	Recent colon cancer surgery (otherwise non-immunocompromised)	Necrotizing bacteremic pneumonia	ICU[1] Support	Deceased (<12 h)
**Goncalves *et al.* [63]**	24/M	None (Athletic); Sea water exposure	Bilateral haemorrhagic necrotizing pneumonia	Standard ICU care	Deceased (within hours)
**Issa *et al.* [70]**	30/M	Trauma; Delayed splenic rupture	Severe bilateral pneumonia	VV-ECMO[2]	Recovered

[1]ICU: Intensive Care Unit. [2]VV-ECMO: venovenous extracorporeal membrane oxygenation.

Table S3. Resistance rates of *Burkholderia cepacia* complex (Bcc) to commonly used antimicrobial agents.

Antimicrobial agent	Class	Resistance rate
Piperacillin-tazobactam	Penicillin/β-lactamase inhibitor	73.6%
Ceftazidime	Third-generation cephalosporin	58.6%
Levofloxacin	Fluoroquinolone	57.4%
Carbapenems	Carbapenem	42.0%
Trimethoprim-Sulphamethoxazole	Sulfonamide/DHFR inhibitor	30.3%
